# Mechanism of the Immunomodulatory Effect of the Combination of Live *Bifidobacterium*, *Lactobacillus*, *Enterococcus*, and *Bacillus* on Immunocompromised Rats

**DOI:** 10.3389/fimmu.2021.694344

**Published:** 2021-06-15

**Authors:** Longxian Lv, Deguang Mu, Yiling Du, Ren Yan, Huiyong Jiang

**Affiliations:** ^1^ State Key Laboratory for the Diagnosis and Treatment of Infectious Diseases, National Clinical Research Center for Infectious Diseases, Collaborative Innovation Center for Diagnosis and Treatment of Infectious Diseases, The First Affiliated Hospital, College of Medicine, Zhejiang University, Hangzhou, China; ^2^ Zhejiang Provincal People’s Hospital, People’s Hospital of Hangzhou Medical College, Hangzhou, China; ^3^ Institute of Pharmaceutical Biotechnology and The First Affiliated Hospital, Zhejiang University School of Medicine, Hangzhou, China

**Keywords:** probiotics, immunomodulation, gut microbiota, metabolome, transcriptome

## Abstract

Immunodeficiency is a very common condition in suboptimal health status and during the development or treatment of many diseases. Recently, probiotics have become an important means for immune regulation. The present study aimed to investigate the mechanism of the immunomodulatory effect of a combination of live *Bifidobacterium*, *Lactobacillus*, *Enterococcus*, and *Bacillus* (CBLEB), which is a drug used by approximately 10 million patients every year, on cyclophosphamide-immunosuppressed rats. Cyclophosphamide (40 mg/kg) was intraperitoneally injected to induce immunosuppression in a rat model on days 1, 2, 3, and 10. Starting from day 4, the rats were continuously gavaged with CBLEB solution for 15 days. The samples were collected to determine routine blood test parameters, liver and kidney functions, serum cytokine levels, gut microbiota, fecal and serum metabolomes, transcriptomes, and histopathological features. The results indicated that CBLEB treatment reduced cyclophosphamide-induced death, weight loss, and damage to the gut, liver, spleen, and lungs and eliminated a cyclophosphamide-induced increase in the mean hemoglobin content and GGT, M-CSF, and MIP-3α levels and a decrease in the red blood cell distribution width and total protein and creatinine levels in the blood. Additionally, CBLEB corrected cyclophosphamide-induced dysbiosis of the gut microbiota and eliminated all cyclophosphamide-induced alterations at the phylum level in rat feces, including the enrichment in Proteobacteria, Fusobacteriota, and Actinobacteriota and depletion of Spirochaetota and Cyanobacteria. Furthermore, CBLEB treatment alleviated cyclophosphamide-induced alterations in the whole fecal metabolome profile, including enrichment in 1-heptadecanol, succinic acid, hexadecane-1,2-diol, nonadecanoic acid, and pentadecanoic acid and depletion of benzenepropanoic acid and hexane. CBLEB treatment also alleviated cyclophosphamide-induced enrichment in serum D-lyxose and depletion of serum succinic acid, D-galactose, L-5-oxoproline, L-alanine, and malic acid. The results of transcriptome analysis indicated that the mechanism of the effect of CBLEB was related to the induction of recovery of cyclophosphamide-altered carbohydrate metabolism and signal transduction. In conclusion, the present study provides an experimental basis and comprehensive analysis of application of CBLEB for the treatment of immunodeficiency.

## Introduction

The immune system is ubiquitous throughout the body and involves a variety of molecules, cells, tissues, and organs (1). The immune system can recognize and remove foreign antigens, such as microbes and pollutants, to resist the attacks by these agent and can distinguish between self and nonself to recognize and remove mutated tumor cells, aging cells, dead cells, and other harmful components of the body at any time, maintaining a stable internal environment through autoimmune tolerance and immune regulation (2). However, immune dysfunction may be induced by various conditions, such as environmental pollution, unhealthy lifestyle, aging, stress, disease, radiotherapy, and chemotherapy (3). Therefore, recent studies have focused on the strategies that maintain or promote a balance of the immune system to delay aging and reduce incidence of infections, tumors, and other related diseases (4), including specific resistance to the SARS-CoV-2 (severe acute respiratory syndrome coronavirus 2) pathogen causing the pandemic.

The gut microbiota are necessary for the development and maintenance of the human immune system. Deficiencies of the microbiota lead to incomplete development of the immune system (5). Comparison with specific pathogen-free (SPF) mice indicated that germ-free mice manifest an expanded decrease in the number of T cells and other immune cells and impaired antibody secretion and immune responses mediated by these cells (6). Similarly, the immunity of SPF animals, in which the gut microbiota was destroyed using broad spectrum antibiotics, is greatly affected; this influence is partially reflected by a reduction in the numbers of T and B cells in many tissues (5, 6). Correspondingly, many gut microbes have important immunoregulatory functions: segmented filamentous bacteria can induce Th17 cells; *Clostridium* species can cause the accumulation of Foxp3^+^ regulatory T cells; *Bacteroides fragilis* can induce CD4^+^ T cell-dependent and -independent immune responses; and *Bifidobacterium bifidum* strains can synergize with immune checkpoint inhibitors to reduce the tumor burden (7, 8). In this context, probiotics have become an important means for immune regulation, health maintenance, and disease prevention.

Cyclophosphamide is a cytotoxic alkylating agent mainly used for the treatment of stage III and IV malignant lymphomas. Other indications of cyclophosphamide include breast cancer, disseminated neuroblastoma, retinoblastoma, pediatric minimal change nephrotic syndrome, and ovarian adenocarcinoma. However, cyclophosphamide has some side effects, such as hemorrhagic cystitis and alopecia. Moreover, cyclophosphamide is an immunosuppressive agent most commonly used for the treatment of many autoimmune diseases and as immunosuppressive therapy during blood and marrow transplantations (9). Administration of cyclophosphamide can suppress the immune response of modulatory lymphocytes (10). Cyclophosphamide-treated immunosuppressed rats are an animal model often used for evaluation of immunoregulatory effects of immunomodulatory compounds (11–13).

A combination of live *Bifidobacterium*, *Lactobacillus*, *Enterococcus*, and *Bacillus* (CBLEB) is a probiotic drug widely used in China. CBLEB is formulated from *Bifidobacterium infantis* CGMCC0460.1, *Lactobacillus acidophilus* CGMCC0460.2, *Enterococcus faecalis* CGMCC0460.3, and *Bacillus cereus* CGMCC0460.4. The present study aimed to investigate the mechanism of the regulatory effect of CBLEB using a cyclophosphamide-immunosuppressed rat model.

## Methods

### Probiotics

Probiotic powders of *Bifidobacterium infantis* CGMCC0460.1, *Lactobacillus acidophilus* CGMCC0460.2, *Enterococcus faecalis* CGMCC0460.3, and *Bacillus cereus* CGMCC0460.4 were purchased from Hangzhou Yuanda Biopharmaceutical Co., Ltd. Probiotic powders were dissolved in sterile saline to prepare CBLEB solution (*Bifidobacterium infantis*, 1.41 × 10^7^ CFU/mL; *Lactobacillus acidophilus*, 1.41 × 10^7^ CFU/mL; *Enterococcus faecalis*, 1.41 × 10^6^ CFU/mL; and *Bacillus cereus*, 1.41 × 10^5^ CFU/mL) for subsequent use.

### Animal Experiment

Twenty-seven SPF male Sprague-Dawley (SD) rats weighing 200-300 g were randomly divided into three groups of nine rats per group. Rats in the cyclophosphamide model (CTX) group were intraperitoneally injected with cyclophosphamide and gavaged with normal saline. Rats in the CBLEB treatment (CTX+CBLEB) group were intraperitoneally injected with cyclophosphamide and gavaged with CBLEB solution. Rats in the healthy control (HC) group were intraperitoneally injected with normal saline and gavaged with normal saline. The animals were maintained on a 12-h light:12-h dark cycle at room temperature (22 ± 2°C). All animal experiments were reviewed and approved by the Animal Care and Use Committee of the First Affiliated Hospital, School of Medicine, Zhejiang University.

On days 1, 2, 3, and 10, cyclophosphamide (40 mg/kg) was intraperitoneally injected in rats of the CTX and CTX+CBLEB groups, and normal saline was injected in the animals of the HC group. Starting from day 4, the rats in the CTX+CBLEB group were continuously gavaged with CBLEB solution (*Bifidobacterium infantis*, 4.7 × 10^7^ CFU/kg; *Lactobacillus acidophilus*, 4.7 × 10^7^ CFU/kg; *Enterococcus faecalis*, 4.7 × 10^6^ CFU/kg; and *Bacillus cereus*, 4.7 × 10^5^ CFU/kg) for 15 days; rats in the HC and CTX groups were administered normal saline for 15 days. Rats were anesthetized with 400 mg/kg chloral hydrate by intraperitoneal injection on day 19. Blood, liver, spleen, lung, colon, thymus, and fecal samples were collected and immediately used or stored at -80°C until use.

### Routine Blood Tests and Liver and Kidney Function Tests

Routine blood tests, such as white blood cell, neutrophil, lymphocyte, monocyte, eosinophil, and basophil counts (BA), were performed using an XN-2000 automatic hematology analyzer (Sysmex, Tokyo, Japan). Liver and kidney function tests (total protein, albumin, globulin, alanine aminotransferase (ALT), aspartate aminotransferase (AST), alkaline phosphatase (ALP), total bilirubin, direct bilirubin, indirect bilirubin, γ-glutamyltransferase (GGT), creatinine, urea nitrogen, and uric acid) were performed using an automatic biochemical analyzer (Hitachi 7600–210; Tokyo, Japan).

### Detection of Serum Cytokines

The levels of the following 23 cytokines in the serum were determined using a Bio-Plex Pro™ rat cytokine 23-plex assay kit (Bio-Rad Laboratories, Hercules, CA, USA): interleukin-1α (IL-1α), IL-1β, IL-2, IL-4, IL-5, IL-6, IL-7, IL-10, IL-12, IL-13, IL-17A, IL-18, granulocyte colony-stimulating factor (G-CSF), granulocyte-macrophage colony-stimulating factor (GM-CSF), macrophage colony-stimulating factor (M-CSF), monocyte chemoattractant protein 1 (MCP-1), macrophage inflammatory protein 1α (MIP-1α), MIP-3α, tumor necrosis factor-α (TNF-α), interferon-γ (IFN-γ), vascular endothelial growth factor (VEGF), growth-regulated α protein (GRO/KC), and regulated upon activation, normal T-cell expressed and secreted protein (RANTES).

### Amplification, Sequencing, and Analysis of the 16S rDNA V3-V4 Region

Fecal DNA was extracted using a QIAamp fast DNA stool mini kit (Qiagen, Hilden, Germany). The 16S rDNA V3-V4 region was amplified by PCR using the primers 338F (5’-ACTCCTACGGGAGGCAGCAG-3’) and 806R (5’-GGACTACHVGGGTWTCTAAT-3’). PCR was performed under the following conditions: start at 95°C for 5 min; 25 cycles at 94°C for 50 s, 46°C for 1 min, and 72°C for 30 s; and a final extension step at 72°C for 5 min (14, 15). The libraries were prepared for Illumina sequencing using a NEXTflex rapid DNA-seq kit (Bioo Scientific, Austin, TX, USA) according the manufacturer’s instructions. DNA sequencing was performed on the Illumina MiSeq platform (Illumina, San Diego, CA, USA) using the paired-end mode (2 × 300-bp pair ends).

The raw reads were cleaned, filtered and merged using FLASH v1.2.8. Vsearch v2.3.4 was used to select operational taxonomic units (OTUs) with sequence similarity greater than 97%. QIIME (Quantitative Insights Into Microbial Ecology) v1.9.1 was used for OTU clustering, identification based on the Greengenes and NCBI 16S Microbial databases, and subsequent statistical analysis of microbial diversity and differential enrichment.

### Assay of Fecal and Serum Metabolites

The metabolome was assayed as described previously (16, 17). Briefly, 20 mg of feces or 200 μL of the serum was added to 800 μL of precooled chromatography grade methanol (Sigma-Aldrich, St. Louis, MO, USA). The fecal mixture was then homogenized three times using a Precellys Evolution instrument (Bertin Technologies, USA) at 5,000 rpm for 30 s with 15 s intervals between the rounds, and the serum mixture was fully mixed using a vortex. All samples were incubated at 4°C overnight and centrifuged at 14,000 rpm for 15 min; the supernatants were filtered through a 0.22 μm membrane. Subsequently, 20 μL of heptadecanoic acid (1 mg/mL) was added to all filtrates as an internal reference. The samples were dried under nitrogen at room temperature, and 50 μL of methoxamine/pyridine (14 mg/mL) was added. The samples were fully mixed, sealed, and incubated at 37°C for 24 h, and 50 μL of N,O-bis(trimethylsilyl)acetamide containing 1% trimethylchlorosilane was added to the samples. The samples were fully mixed and incubated at 70°C for 2 h. The samples were analyzed using an Agilent 7890A-5975C gas chromatography-mass spectrometry (GC-MS) system (Agilent, USA) as described previously. Metabolites were identified using the NIST 17 resources, and the data were analyzed using SIMCA software (v14.1).

### Extraction of Tissue RNA and Transcriptome Analysis

Total RNA was extracted from the spleen and colon using an RNeasy Plus Mini kit (Qiagen, Valencia, CA, USA). The library was constructed using a NEBNext^®^ Ultra™ RNA library prep kit (Illumina). cDNA (250-300 bp) was screened using an AMPure XP system (Beckman Coulter, Beverly, MA, USA). Preliminary quality of the constructed library was determined using a Qubit2.0 fluorometer (Invitrogen, Carlsbad, CA, USA); the insert size was verified using an Agilent 2100 bioanalyzer (Agilent Technologies, CA, USA), and effective concentration was quantified by qRT-PCR using a QuantStudio 12K Flex real-time PCR system (Thermo Fisher Scientific, USA). Sequencing was performed on an Illumina NovaSeq 6000 (Illumina, San Diego, CA, USA) using the paired-end mode (2 × 150 bp). The raw data were trimmed using Trimmomatic to generate the clean data (18). HISAT2 v2.0.5 was used to generate an index of the reference genomes and to align paired-end clean reads with a reference genome. StringTie (1.3.3b) was used to assemble novel transcripts. The expression value (fragments per kilobase of transcripts per million mapped fragments, FPKM) of each gene was calculated using FeatureCounts (1.5.0-p3). DESeq2 (1.16.1) was used to analyze differential expression between two groups. ClusterProfiler (3.4.4) was used to analyze statistical enrichment in differentially expressed genes of the Kyoto Encyclopedia of Genes and Genomes (KEGG) pathways.

### RT-qPCR Analysis

Some representative genes detected by transcriptome analysis were validated by reverse transcription quantitative PCR (RT-qPCR). Total RNA was extracted from the spleen and colon using an RNeasy Plus Mini kit (Qiagen, Valencia, CA, USA). Total RNA was converted into cDNA using a PrimeScript™ RT reagent kit (Takara Biomedicals, Kusatsu, Japan) and assayed by RT-qPCR with Premix Ex Taq (Takara Biomedicals, Kusatsu, Japan) on a ViiA7 real-time PCR system (Applied Biosystems, Waltham, Massachusetts, USA). The primer sequences for the indicated genes are provided in **Supplementary Table 3**. The housekeeping gene glyceraldehyde-3-phosphate dehydrogenase (GAPDH) was used as an internal control. Transcription of the genes was recalculated into relative expression normalized against internal control, and the data were used in subsequent analysis.

### Histopathological Evaluation

The liver, spleen, lung, and colon tissues (0.5 × 0.5 × 0.5 cm) were fixed in 10% neutral formaldehyde solution, dehydrated, embedded in paraffin wax, cut into sections, and stained using hematoxylin and eosin (H&E). The sections were scanned with a section scanner, and the images were used to evaluate the degree of tissue damage, inflammation, and necrosis in the liver, spleen, lung, and colon. Hepatic injuries were assessed based on the histological activity index (HAI) (15). Intestinal mucosal injury was graded from 0 to 5 points (14). Pulmonary histological damage was quantified using the American Thoracic Society 2010 Lung Injury Scoring System (19). The pathological severity score of the spleen was graded from 0 to 3 points as described previously (20).

### Statistics

The following parameters were compared between the groups: body weight, spleen index, thymus index, routine blood biomarkers, liver and kidney functions, serum cytokine levels, α-diversity of the gut microbiota, and fecal metabolites. The Kolmogorov-Smirnov test was used to determine whether the data are normally distributed within a group. Subsequently, one-way ANOVA was used to compare normally distributed data; otherwise, the Mann-Whitney U test was used for comparison. The Wilcoxon rank sum test combined with the Benjamini-Hochberg method was used to compare relative abundances of intestinal bacteria between various groups. The data are expressed as the mean ± SEM (standard error of the mean) unless specified otherwise. A two-tailed *P* value or *P*_adj_ less than 0.05 was considered statistically significant.

## Results

### CBLEB Reduces Cyclophosphamide-Induced Death, Weight Loss, Immune Dysfunction, and Organ Damage in Rats

**Figure 1:**
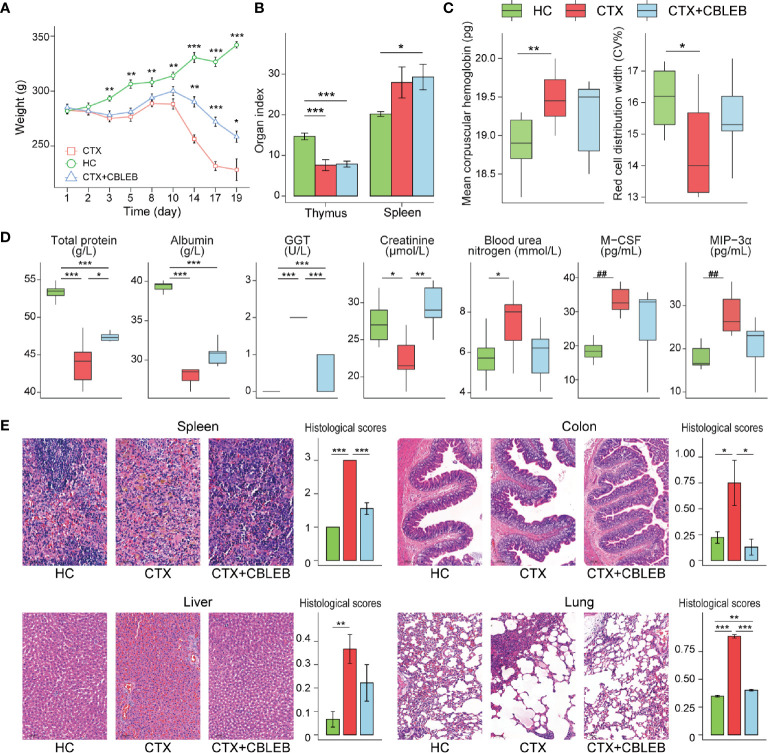
CBLEB reduces cyclophosphamide-induced death, weight loss, immune dysfunction, and organ damage. **(A)** Weight plot (*P < 0.05; **P < 0.01; ***P < 0.001 *versus* the CTX group), **(B)** thymus and spleen indexes, **(C)** mean corpuscular hemoglobin and red cell distribution width in the serum samples, **(D)** concentrations of total protein, albumin, GGT, creatinine, blood urea, M-CSF, and MIP-3α in the serum samples; **(E)** representative images of the spleen, colon, liver, and lung samples stained by H&E and the corresponding histological scores in the CTX (n = 6), CTX+CBLEB (n = 9), and HC (n = 9) groups. *P < 0.05; **P < 0.01; ***P < 0.001; ^##^ *P*_adj_ < 0.01.

During the experiment, the body weight of rats in the HC group was steadily increased. However, intraperitoneal injection of cyclophosphamide induced weight loss in rats. The body weight of rats in the CTX group was significantly lower than that of rats in the HC group starting from day 3, and the body weight was decreased more rapidly after fourth intraperitoneal injection of cyclophosphamide on day 10. Intragastric administration of CBLEB significantly reduced the weight loss induced by cyclophosphamide in rats (**Figure 1A**). At the end of the experiment, three (33%) rats in the CTX group died; however, no rats died in the HC and CTX+CBLEB groups. These results indicated that CBLEB can alleviate cyclophosphamide-induced death and weight loss.

Intragastric administration of CBLEB suppressed a cyclophosphamide-induced increase in the mean hemoglobin content and a decrease in the red blood cell distribution width; as a result, the differences in these two indexes between the CTX+CBLEB and HC groups were not significant (**Figure 1C**). Furthermore, intragastric administration of CBLEB significantly reduced a cyclophosphamide-induced decrease in total protein, albumin, and creatinine and an increase in GGT levels (**Figure 1D**). Additionally, treatment with CBLEB eliminated a cyclophosphamide-induced increase in serum urea nitrogen (**Figure 1D**). The results of serum cytokine assays indicated that intraperitoneal injection of cyclophosphamide induced an increase in the serum levels of M-CSF (*P*_adj_ = 1.57E-03) and MIP-3α (*P*_adj_ = 2.96E-03), and these changes were reversed by CBLEB treatment. (**Figure 1D**).

CBLEB alleviated organ damage induced by cyclophosphamide. The thymus index in the CTX and CTX+CBLEB groups was significantly lower than that in the HC group (**Figure 1B**), indicating that cyclophosphamide has a strong immunosuppressive effect. The spleen index in the CTX+CBLEB group was significantly higher than that in the HC group (**Figure 1B**). This finding may be due to compensatory enlargement of the spleen caused by CBLEB under immunodeficient conditions, and this compensatory effect may improve the host immunity. The data of H&E staining revealed hemosiderin deposition in the spleen in the CTX group, but not in the CTX+CBLEB group, and significant alleviation of splenic congestion was observed in the CTX+CBLEB group compared with congestion detected in the CTX group (**Figure 1E**). The villi and crypts of the colon were arranged tightly and neatly in the HC group but were partially destroyed in the CTX group, and the number of colonic glands was decreased in the CTX group. CBLEB treatment did not induce detectable colon injury (**Figure 1E**). Significant vascular damage was observed in the liver in the CTX group but was not detected in the CTX+CBLEB group (**Figure 1E**). Inflammatory exudations and a large number of plasma cells were observed in the lungs in the CTX group. CBLEB treatment decreased both lung injury and inflammatory cell infiltration (**Figure 1E**). The histological scores of the liver, colon, lung, and spleen were significantly higher in the CTX group than those in the HC group. CBLEB treatment significantly lowered the histological scores in the colon, lung, and spleen (**Figure 1E**).

### CBLEB Improves Cyclophosphamide-Induced Dysbiosis of the Gut Microbiota

**Figure 2:**
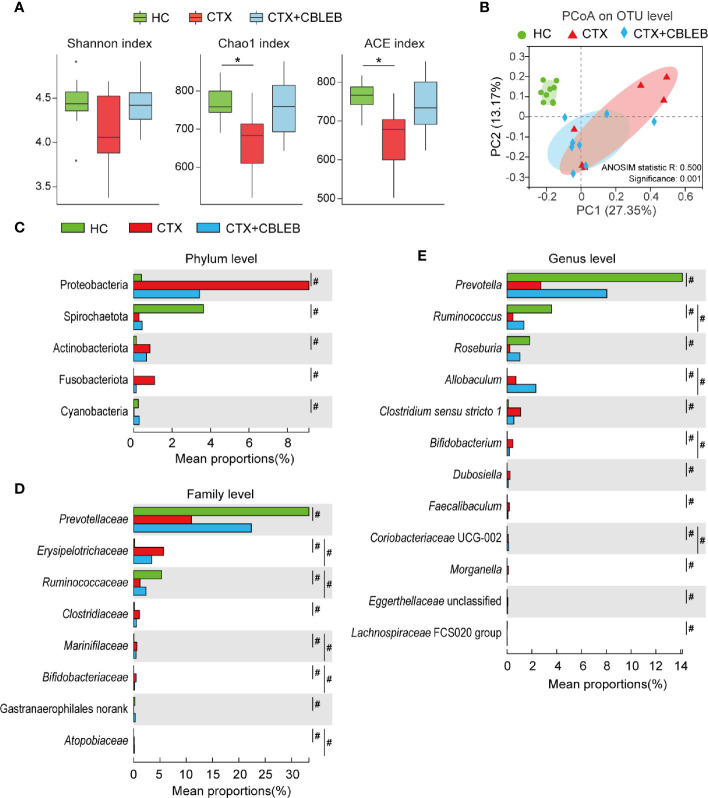
CBLEB reverses cyclophosphamide-induced dysbiosis of the gut microbiota. **(A)** Box plot of species richness and flora diversity estimated based on the Chao1, ACE, and Shannon indexes. **(B)** Two-dimensional PCoA plot based on the Bray-Curtis matrix confirmed by ANOSIM. **(C–E)** Alterations in relative abundance of bacterial taxa in the CTX (n = 6), CTX+CBLEB (n = 8), and HC (n = 9) groups at the phylum **(C)**, family **(D)**, and genus **(E)** levels. *P < 0.05; ^#^ *P*_adj_ < 0.05.

No significant differences in the Shannon index were observed between the HC, CTX, and CTX+CBLEB groups, indicating similar community diversity in these groups (**Figure 2A**). The Chao1 and ACE (abundance-based coverage estimator) indexes in the CTX group were significantly lower than those in the HC group, indicating that the richness of fecal microbiota in rats was significantly reduced after cyclophosphamide administration. Intragastric administration of CBLEB reversed a cyclophosphamide-induced reduction in the Chao1 index, and the index was not different between the CTX+CBLEB and HC groups (**Figure 2A**). The results of principal coordinate analysis (PCoA) indicated that the fecal microbiota profiles in the HC, CTX, and CTX+CBLEB groups formed three clusters due to significant differences in the composition of fecal microbiota between these groups (**Figure 2B**). This result was confirmed by ANOSIM (analysis of similarity) (*P* = 0.001).

Intragastric administration of CBLEB greatly reduced the changes in fecal bacterial taxa caused by cyclophosphamide. First, the results of phylum-level analysis indicated that cyclophosphamide induced enrichment in Proteobacteria, Fusobacteriota, and Actinobacteriota and depletion of Spirochaetota and Cyanobacteria in rat feces, and CBLEB treatment eliminated cyclophosphamide-induced alterations in all these phyla (**Figure 2C**). Second, at the family level, cyclophosphamide induced enrichment in *Erysipelotrichaceae*, *Clostridiaceae*, *Marinifilaceae*, *Bifidobacteriaceae*, and *Atopobiaceae* and depletion of *Prevotellaceae*, *Ruminococcaceae*, and Gastranaerophilales (unranked) in rat feces. CBLEB treatment eliminated enrichment in *Clostridiaceae* and depletion of *Prevotellaceae* and Gastranaerophilales (unranked) induced by cyclophosphamide (**Figure 2D**). Third, the results of genus-level analyses indicated that cyclophosphamide induced enrichment in *Clostridium sensu stricto* 1, *Allobaculum*, *Bifidobacterium*, *Dubosiella*, *Faecalibaculum*, *Morganella*, *Coriobacteriaceae* UCG-002, and *Eggerthellaceae* unclassified and depletion of *Prevotella*, *Ruminococcus*, *Roseburia*, and *Lachnospiraceae* FCS020 group in rat feces. CBLEB treatment eliminated cyclophosphamide-induced enrichment in *Clostridium sensu stricto* 1, *Dubosiella*, *Faecalibaculum*, *Morganella*, and *Eggerthellaceae* unclassified and depletion of *Prevotella*, *Roseburia*, and *Lachnospiraceae* FCS020 group (**Figure 2E**).

### CBLEB Alleviates Cyclophosphamide-Induced Disorder of Gut Metabolism in Rats

**Figure 3:**
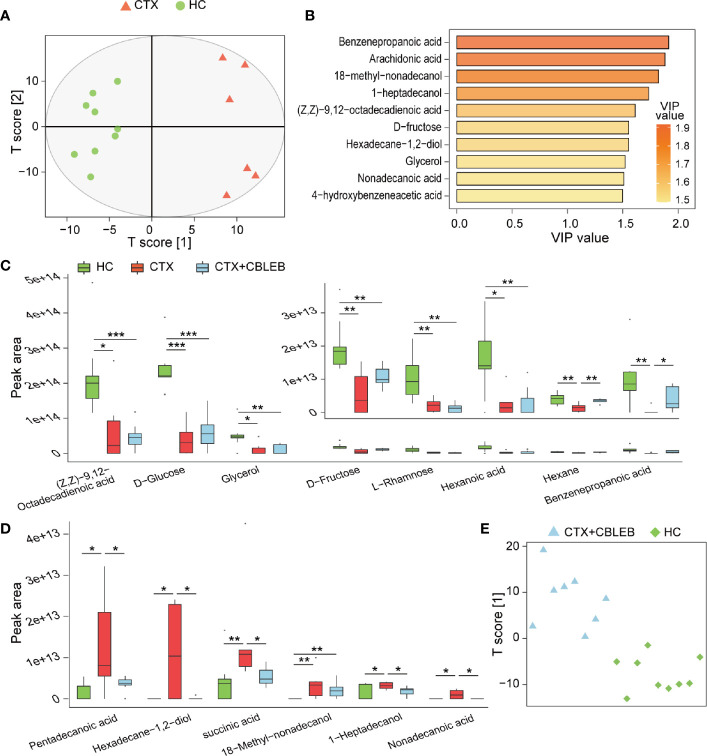
CBLEB alleviates cyclophosphamide-induced gut metabolism disorder. **(A)** OPLS-DA plot illustrating clear separation of gut metabolic profiles of the CTX (n = 6) and HC (n = 9) groups. **(B)** VIP values of 10 metabolites with the highest contribution to the differences between the CTX and HC groups. **(C)** Levels of eight metabolites reduced in the CTX group compared with those in the HC group. **(D)** Levels of six metabolites that were increased in the CTX group compared with those in the HC group. **(E)** OPLS-DA plot illustrating that gut metabolic profiles of the CTX+CBLEB (n = 8) group were in distinguishable from those of the HC (n = 9) group. (*P < 0.05; **P < 0.01; ***P < 0.001).

Investigation of therapeutic effect of CBLEB on cyclophosphamide-injected rats using GC/MS identified a total of 112 fecal metabolites. The data of the orthogonal projections to latent structures discriminant analysis (OPLS-DA) plot indicated that the samples of the CTX group were clustered separately from the samples of the HC group, indicating that metabolome profiles of the two groups were completely different (**Figure 3A**). Analysis using variable influence on projection (VIP) values higher than 1.5 as a threshold demonstrated that various metabolites were very important for discrimination of the CTX group from the HC group by the OPLS-DA model; these metabolites included benzenepropanoic acid, arachidonic acid, 18-methyl-nonadecanol, 1-heptadecanol, (Z,Z)-9,12-octadecadienoic acid, D-fructose, hexadecane-1,2-diol, glycerol, nonadecanoic acid, and 4-hydroxybenzeneacetic acid (**Figure 3B**).

Subsequently, we explored the differences in individual fecal metabolite between the CTX and HC groups. On the one hand, intraperitoneal injection of cyclophosphamide induced depletion of eight fecal metabolites (benzenepropanoic acid, (Z,Z)-9,12-octadecadienoic acid, hexanoic acid, glycerol, hexane, D-fructose, D-glucose, and L-rhamnose), suggesting that cyclophosphamide treatment may induce a reduction in the production or an increase in demand in the case of these compounds (**Figure 3C**). On the other hand, intraperitoneal injection of cyclophosphamide caused enrichment in six fecal metabolites (1-heptadecanol, 18-methyl-nonadecanol, succinic acid, hexadecane-1,2-diol, nonadecanoic acid, and pentadecanoic acid), indicating that cyclophosphamide treatment may induce an increase in the production or a decrease in utilization in the case of these compounds (**Figure 3D**).

OPLS-DA analysis was unable to distinguish fecal samples of the CTX+CBLEB group from those of the HC group, indicating that the overall metabolome profiles of the two groups were similar (**Figure 3E**). In the case of individual metabolites, CBLEB alleviated cyclophosphamide-induced enrichment in 1-heptadecanol, succinic acid, hexadecane-1,2-diol, nonadecanoic acid, and pentadecanoic acid in rat feces on the one hand; the levels of these five compounds in the CTX+CBLEB group were significantly lower than those in the CTX group (**Figure 3D**). On the other hand, CBLEB alleviated cyclophosphamide-induced depletion of benzenepropanoic acid and hexane; the levels of these two compounds in the CTX+CBLEB group were significantly higher than those in the CTX group (**Figure 3C**). These results indicated that intragastric administration of CBLEB can be used to treat cyclophosphamide-induced gut metabolic disorder in rats.

### CBLEB Alleviates Cyclophosphamide-Induced Disorder of Serum Metabolism in Rats

**Figure 4:**
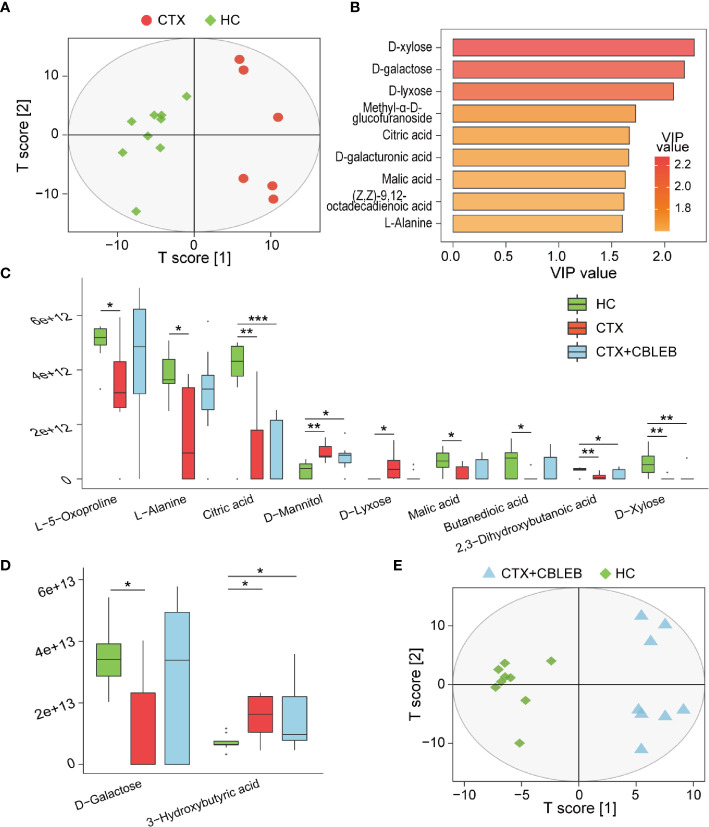
CBLEB alleviates cyclophosphamide-induced serum metabolism disorders. **(A)** OPLS-DA plot illustrating clear separation of serum metabolic profiles of the CTX (n = 6) and HC (n = 9) groups. **(B)** VIP values of nine metabolites with the highest contribution to the differences between the CTX and HC groups. **(C)** Metabolites depleted after cyclophosphamide treatment and changes in their levels after CBLEB treatment. **(D)** Metabolites enriched after cyclophosphamide treatment and changes in their levels after CBLEB treatment. **(E)** OPLS-DA plot illustrating clear separation of serum metabolic profiles of the CTX+CBLEB (n = 8) and HC groups (n = 9). (*P < 0.05; **P < 0.01; ***P < 0.001).

GC/MS-based investigation of the therapeutic mechanism of CBLEB in cyclophosphamide-injected rats identified a total of 60 metabolites in the serum. The data of OPLS-DA indicated that the samples of the CTX and HC groups are clearly divided into two clusters, indicating that metabolome profiles of these two groups were different (**Figure 4A**). A threshold VIP value higher than 1.5 was used to identify nine metabolites (D-xylose, D-galactose, D-lyxose, methyl-alpha-D-glucofuranoside, citric acid, D-galacturonic acid, malic acid, (Z,Z)-9,12-octadecadienoic acid, and L-alanine) important for the OPLS-DA model (**Figure 4B**). We then compared the levels of individual serum metabolite between the CTX and HC groups. Intraperitoneal injection of cyclophosphamide induced depletion of 2,3-dihydroxybutanoic acid, citric acid, D-galactose, D-xylose, malic acid, succinic acid, L-5-oxoproline, and L-alanine and enrichment in D-lyxose, D-mannitol, and 3-hydroxybutyric acid in rat serum (**Figures 4C, D**).

The metabolome profile of the CTX+CBLEB group was clustered separately from that of the HC group, indicating that CBLEB treatment did not completely reverse cyclophosphamide-induced alterations in the serum metabolome profile in rats (**Figure 4E**). However, CBLEB treatment alleviated cyclophosphamide-induced enrichment in D-lyxose and depletion of succinic acid, D-galactose, L-5-oxoproline, L-alanine, and malic acid, indicating potential regulation of metabolism of cyclophosphamide-injected rats by treatment with CBLEB (**Figures 4C, D**).

### CBLEB Partially Recovers Cyclophosphamide-Induced Alterations in Metabolism and Signal Transduction Pathways in the Spleen and Colon

Intraperitoneal injection of cyclophosphamide increased the levels of the transcripts of 3,306 genes and decreased the levels of the transcripts of 2,794 genes in the spleen (*P*_adj_ < 0.05). The results of KEGG pathway analysis indicated that cyclophosphamide induced downregulation of 33 signaling pathways, including 10 pathways of the immune system (e.g., T cell receptor signaling pathway, Th1 and Th2 cell differentiation, Th17 cell differentiation, B cell receptor signaling pathway, and Toll-like receptor signaling pathway); seven signal transduction pathways were downregulated, including the NF-kappa B, MAPK, and TNF signaling pathways. Some other important pathways were also downregulated, including PD-L1 expression and the PD-1 checkpoint pathway related to cancer (**Supplementary Table 1**). Moreover, 30 signaling pathways were upregulated by cyclophosphamide treatment, including seven metabolic pathways (e.g., glycolysis/gluconeogenesis, the pentose phosphate pathway, starch and sucrose metabolism, and oxidative phosphorylation), seven human disease pathways (e.g., Parkinson’s disease, Huntington’s disease, and Alzheimer’s disease), four cell growth and death pathways (e.g., the cell cycle and p53 signaling pathway), four replication and repair pathways (e.g., DNA replication and homologous recombination), and other critical pathways (e.g., the cell cycle and DNA replication) (**Supplementary Table 1**).

**Figure 5:**
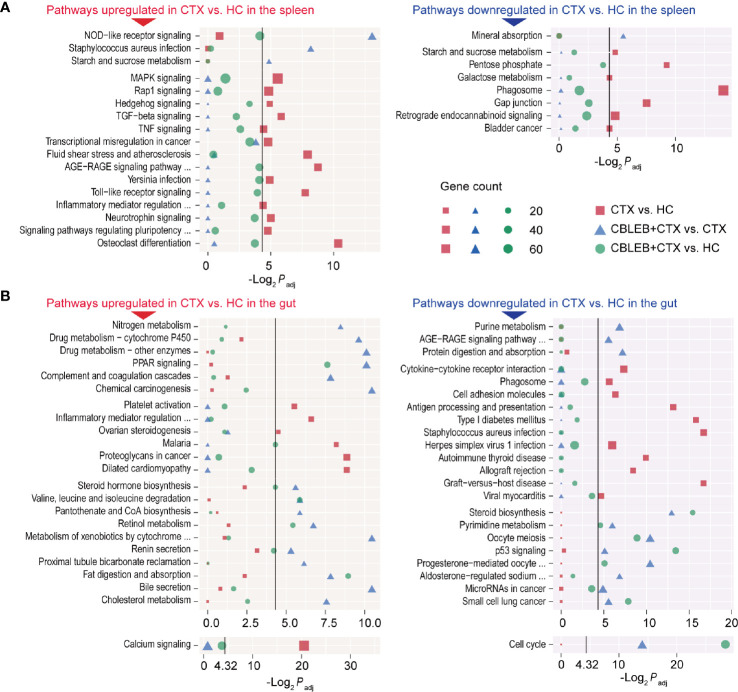
Pathways altered by cyclophosphamide and alleviated by CBLEB in the spleen and colon. **(A)** Pathways upregulated or downregulated by cyclophosphamide and alleviated by CBLEB in the spleen. **(B)** Pathways upregulated or downregulated by cyclophosphamide and alleviated by CBLEB in the colon. (n = 3 in all groups; black vertical lines represent -Log_2_ *P*_adj_ = 4.32 corresponding to *P*_adj_ = 0.05).

Intragastric administration of CBLEB induced significant downregulation of the NOD-like receptor signaling pathways, *Staphylococcus aureus* infection, and starch and sucrose metabolism and upregulation of the mineral absorption pathway in the spleen of cyclophosphamide-injected rats (**Figure 5A**). Furthermore, CBLEB reversed downregulation of 14 signaling pathways caused by cyclophosphamide, including five signal transduction pathways (the MAPK, Rap1, Hedgehog, TGF-beta, and TNF pathways), four disease-related signaling pathways (transcriptional misregulation in cancer, fluid shear stress and atherosclerosis, the AGE-RAGE signaling pathway in diabetic complications, and Yersinia infection), and some other important pathways (e.g., the Toll-like receptor signaling pathway) (**Figure 5A**). Moreover, CBLEB abolished cyclophosphamide-induced upregulation of seven signaling pathways, including three metabolism-related signaling pathways (the pentose phosphate, galactose metabolism, and phagosome pathways) and pathways related to gap junctions, retrograde endocannabinoid signaling, and bladder cancer (**Figure 5A**). These results indicated that CBLEB plays an important role in the maintenance of immunity, metabolism, and important life processes in cyclophosphamide-injected rats.

Intraperitoneal injection of cyclophosphamide induced an increase in the levels of the transcripts of 350 genes and a decrease in the levels of the transcripts of 414 genes in the rat colon. The results of KEGG analysis indicated that cyclophosphamide induced downregulation of 22 signaling pathways in the colon, including three signal transduction pathways (PI3K-Akt, calcium, and phospholipase D), nine disease-related signaling pathways (some of which are specifically associated with parasite infections, e.g., African trypanosomiasis, amoebiasis, and malaria), three immune pathways (intestinal immune network for IgA production, hematopoietic cell lineage, and platelet activation), two digestive pathways (pancreatic secretion and protein digestion and absorption), and other important pathways (e.g., focal adhesion) (**Supplementary Table 2**). Moreover, cyclophosphamide induced upregulation of 25 signaling pathways in the colon tissue, including four immune pathways (complement and coagulation cascades, NOD-like receptor signaling pathway, antigen processing and presentation, and cytosolic DNA-sensing pathway), 14 disease-related pathways (some of which are specifically associated with eight bacterial infection and viral pathways, e.g., *Staphylococcus aureus* infection, pertussis, influenza A, and hepatitis C), and other important signaling pathways (e.g., nitrogen metabolism) (**Supplementary Table 2**).

Intragastric administration of CBLEB alleviated cyclophosphamide-induced disturbances of the signaling pathways in the colon. First, CBLEB significantly reduced nitrogen metabolism, drug metabolism-cytochrome P450, drug metabolism-other enzymes, PPAR signaling pathway, complement and coagulation cascades, and chemical carcinogenesis and significantly upregulated purine metabolism, the AGE-RAGE signaling pathway in diabetic complications, and protein digestion and absorption (**Figure 5B**). Second, CBLEB reversed cyclophosphamide-induced alterations in some pathways in the colon; these pathways were different between the CTX and HC groups but not between the CTX+CBLEB and HC groups. These cyclophosphamide-induced alterations reversed by CBLEB included downregulation of the calcium signaling pathway, platelet activation, inflammatory mediator regulation of TRP channels, ovarian steroidogenesis, malaria, proteoglycans in cancer, dilated cardiomyopathy, upregulation of cytokine-cytokine receptor interaction, phagosomes, cell adhesion molecules, antigen processing and presentation, type I diabetes mellitus, *Staphylococcus aureus* infection, herpes simplex virus 1 infection, autoimmune thyroid disease, allograft rejection, graft-*versus*-host disease, and viral myocarditis (**Figure 5B**). Third, CBLEB altered some pathways in the colon of cyclophosphamide-injected rats; however, the corresponding differences between the CTX and HC groups were not significant. CBLEB predominantly downregulated steroid hormone biosynthesis, valine, leucine, and isoleucine degradation, pantothenate and CoA biosynthesis, retinol metabolism, metabolism of xenobiotics by cytochrome P450, renin secretion, proximal tubule bicarbonate reclamation, fat digestion and absorption, bile secretion, and cholesterol metabolism and upregulated steroid biosynthesis, pyrimidine metabolism, the cell cycle, oocyte meiosis, the p53 signaling pathway, progesterone-mediated oocyte maturation, aldosterone-regulated sodium reabsorption, microRNAs in cancer, and small cell lung cancer (**Figure 5B**).

To validate the results of transcriptome assays, we performed RT-qPCR analysis of representative genes in the colon (*Tlr4* and *Tlr5*) and spleen (*Nf-κb* and *Tjp1*). The data indicated that the expression of these genes was similar to the levels obtained by transcriptome analysis (**Supplementary Figure 1**).

#### Associations of CBLEB-Influenced Fecal Bacteria, Fecal and Serum Metabolites, and Gut and Spleen Genes

**Figure 6:**
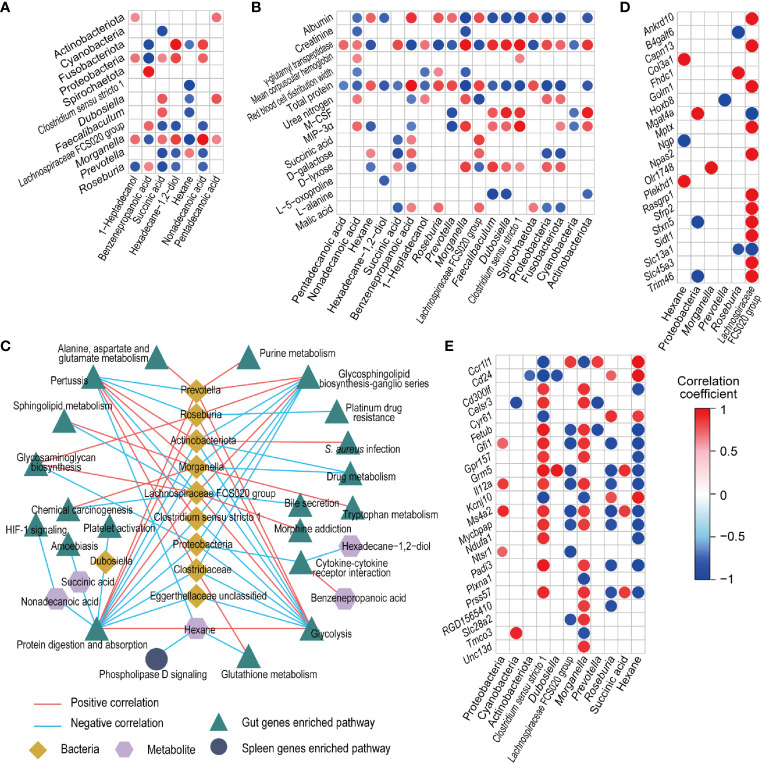
Associations between fecal bacteria, fecal and serum metabolites, and gut and spleen genes influenced by CBLEB. **(A)** Correlation of CBLEB-influenced fecal bacteria with fecal metabolites. **(B)** Correlation of CBLEB-influenced fecal bacteria and metabolites with blood cytokines, metabolites, and indicators of the liver and kidney functions. **(C)** Pathways identified based on the genes that were positively or negatively correlated with individual bacteria or metabolites according to the KEGG database analysis (*P* < 0.01). The correlation coefficients for individual bacteria or metabolites within the corresponding pathway are presented as the mean correlation coefficient of all genes in the pathway positively or negatively correlated with individual bacteria or metabolites. **(D)** Correlation of CBLEB-influenced fecal bacteria and metabolites with the gut genes (*P*_adj_ < 0.01). **(E)** Correlation of CBLEB-influenced fecal bacteria and metabolites with the spleen genes (*P*_adj_ < 0.05).

The levels of fecal bacteria and metabolites, which were altered by cyclophosphamide and reversed by CBLEB, were substantially correlated with each other. Primarily, the levels of 1-heptadecanol, succinic acid, hexadecane-1,2-diol, nonadecanoic acid, and pentadecanoic acid were positively correlated with the levels of at least one representative of Actinobacteriota, Fusobacteriota, Proteobacteria, *Morganella*, *Clostridium sensu stricto* 1, *Dubosiella*, and *Faecalibaculum* and negatively correlated with the levels of at least one representative of Spirochaetota, Cyanobacteria, *Lachnospiraceae* FCS020 group, *Prevotella*, and *Roseburia*. However, the correlations of the levels of these bacterial taxa with fecal levels of benzenepropanoic acid and hexane were opposite to correlations with other metabolites described above (**Figure 6A**).

The levels of certain fecal microbiota and metabolites altered by cyclophosphamide and reversed by CBLEB were associated with some blood cytokines, metabolites, and indicators of the liver and kidney functions. Serum levels of GGT, the inflammatory cytokines M-CSF and MIP-3α, mean corpuscular hemoglobin, urea nitrogen, and D-lyxose were positively correlated with at least one representative of fecal Actinobacteriota, Proteobacteria, Fusobacteriota, *Clostridium sensu stricto* 1, *Morganella*, *Dubosiella*, and *Faecalibaculum* and with at least one of the fecal levels of succinic acid, 1-heptadecanol, and nonadecanoic acid. In contrast, the levels of total serum protein, albumin, red blood cell distribution width, D-galactose, L-alanine, and malic acid were negatively correlated with at least one representative of fecal Spirochaetota, Cyanobacteria, *Prevotella*, *Roseburia*, and *Lachnospiraceae* FCS020 group and with at least one of the fecal levels of pentadecanoic acid, succinic acid, 1-heptadecanol, nonadecanoic acid, and hexadecane-1,2-diol (**Figure 6B**).

More than 600 correlations were detected at *P*_ajdust_ < 0.05 between fecal bacteria, metabolites, and gut genes whose levels were changed by cyclophosphamide and reversed by CBLEB. Genes that were positively or negatively correlated with individual bacteria or metabolites were used for KEGG pathway analysis (**Figure 6C**). The results indicated that all groups of the gut genes, which were positively correlated with fecal levels of hexane, *Lachnospiraceae* FCS020 group, *Prevotella*, or *Roseburia* and negatively correlated with fecal levels of *Clostridiaceae*, *Clostridium sensu stricto* 1, *Dubosiella*, *Morganella*, Actinobacteriota, Proteobacteria, nonadecanoic acid, or succinic acid, were mapped to the protein digestion and absorption pathway at a suitable significance level. The levels of the gut genes, which were positively correlated with the fecal levels of succinic acid, and all groups of the gut genes, which were negatively correlated with fecal levels of *Clostridiaceae*, *Clostridium sensu stricto* 1, *Morganella*, Actinobacteriota, or Proteobacteria, were mapped to the glycosphingolipid biosynthesis-ganglio series pathway at a suitable significance level. All groups of the gut genes, which were positively correlated with the levels of fecal hexane, *Lachnospiraceae* FCS020 group, *Prevotella*, and *Roseburia* and negatively correlated with the fecal levels of *Clostridiaceae*, *Clostridium sensu stricto* 1, *Morganella*, Actinobacteriota, and Proteobacteria, were mapped to the glycolysis pathway at a suitable significance level. All groups of gut genes, which were positively correlated with fecal levels of *Clostridiaceae*, *Clostridium sensu stricto* 1, *Morganella*, or Actinobacteriota, were mapped to the pertussis pathway. All groups of the gut genes, which were negatively correlated with fecal levels of hexadecane-1,2-diol and Proteobacteria, were mapped to the cytokine-cytokine receptor interaction pathway at a suitable significance level. *P*_ajdust_ < 0.01 was used as the threshold to assess correlations between fecal bacteria or metabolites and the gut genes, including positive correlations of *Lachnospiraceae* FCS020 group with *Trim46* and *Slc13a1* and negative correlations of hexane with *Col3a1* and *Plekhd1* (**Figure 6D**).

At *P*_ajdust_ < 0.05, the levels of certain fecal bacteria (*Morganella* and *Clostridium sensu stricto* 1) were positively correlated with certain spleen genes (*Il12a* and *Ms4a2*) and negatively correlated with other spleen genes (*Cd24* and *Ccr1l1*). In contrast, the levels of certain fecal bacteria (*Lachnospiraceae* FCS020 group and *Roseburia*) were negatively correlated with certain spleen genes (*Il12a* and *Ms4a2*). Additionally, the level of hexane was negatively correlated with certain spleen genes (*Fetub*), and positively correlated with other genes, such as *Ccr1l1* and *Cd24* (**Figure 6E**). Only those spleen genes correlated with gut bacteria or metabolites, which were also negatively correlated with hexane, were significantly enriched in the phospholipase D signaling pathway according to the data of the KEGG pathway analysis (**Figure 6C**).

## Discussion

Immunodeficiency is very common in suboptimal health status and during the development and/or treatment of some diseases. The mechanisms of interactions between the gut microbiota and immune deficiency are attracting increasing attention. The topics include the regulation of the gut microbiota to prevent the occurrence of and deterioration due to immunodeficiency, which have become important concepts in health promotion and disease prevention. CBLEB is a probiotic drug used by approximately 10 million patients every year to treat diarrhea, constipation, and functional dyspepsia. The results of the present study indicated that oral administration of CBLEB in a cyclophosphamide-injected rat model can reduce the damage to the spleen, liver, lung, and colon, alleviate an increase in the serum levels of M-CSF and MIP-3α, restore homeostasis of the gut microbiota and metabolome, and reduce dysbiosis of the serum metabolome. The mechanism underlying the effect of CBLEB is related to the regulation of immune, metabolic, and signal transduction pathways.

The results of the present study indicated that intragastric administration of CBLEB reduced the levels of inflammation and immune injury induced by cyclophosphamide in rats. First, CBLEB reversed a cyclophosphamide-induced increase in the mean hemoglobin content and a decrease in the red blood cell distribution width. This result indicated that CBLEB may contribute to the treatment of cyclophosphamide-induced myelosuppressive anemia and abnormalities of innate immunity related to hemoglobin and red blood cells (21). Second, CBLEB attenuated a cyclophosphamide-induced increase in the serum levels of M-CSF and MIP-3α. M-CSF and MIP-3α are the cytokines related to monocytes and macrophages (22, 23). Alleviation of the changes in M-CSF and MIP-3α by CBLEB indicated that this drug can reduce cyclophosphamide-related inflammation and infections. Third, the data of spleen transcriptome analysis revealed that cyclophosphamide significantly downregulated immune pathways (T cell receptor signaling pathway, Th1 and Th2 cell differentiation, Th17 cell differentiation, B cell receptor signaling pathway, and Toll-like receptor signaling pathway), indicating that cyclophosphamide acts *via* an immunosuppressive mechanism. Intragastric administration of CBLEB alleviated cyclophosphamide-induced downregulation of the Toll-like receptor and NOD-like receptor signaling pathways, which partially reflects the mechanism of immune regulation by CBLEB (24, 25).

The results of the present study indicated that CBLEB can restore cyclophosphamide-induced dysbiosis of the gut microbiota. The data demonstrated enrichment in Proteobacteria members and depletion of Bacteroidetes (*Prevotellaceae*) and Firmicutes (*Lachnospiraceae* and *Ruminococcaceae*) in the feces of cyclophosphamide-injected rats. This finding is consistent with previous reports on cyclophosphamide-induced alterations in gut microbiota (26–30) and may result from cyclophosphamide-induced immunodeficiency because the results of the present study indicated that transcription of the components of the intestinal immune network for immunoglobulin A (IgA) production, hematopoietic cell lineages, and platelet activation was downregulated in cyclophosphamide-injected rats. In return, dysbiosis of the gut microbiota may further impair the health of the host. The levels of certain representative of *Lachnospiraceae* or *Ruminococcaceae* were reduced by cyclophosphamide, and these bacteria can produce short-chain fatty acids, particularly butyrate and propionate; *Roseburia* is a well-known butyrate-producing bacterium, and a decrease in *Roseburia* is considered a sign of dysbiosis in patients with ulcerative colitis (31). The levels of some representatives of Proteobacteria were enhanced by cyclophosphamide, and these bacteria are opportunistic pathogens (32). Similarly, the results of the present study indicated that various immune pathways (NOD-like receptor signaling pathway, antigen processing and presentation, complement and coagulation cascades, and cytosolic DNA-sensing pathway) and several infection-related pathways (*Staphylococcus aureus* infection, pertussis and influenza A) were upregulated by cyclophosphamide. Furthermore, the levels of the gut genes were positively correlated with the levels of Proteobacteria or negatively correlated with *Roseburia*, and these genes were mapped to the protein digestion and absorption pathway at a suitable significance level. CBLEB treatment reversed most of cyclophosphamide-induced alterations in the gut microbiota and cyclophosphamide-induced downregulation of platelet activation and upregulation of antigen processing and presentation, the complement and coagulation cascades, and infection-related pathways, such as *Staphylococcus aureus* infection, indicating that CBLEB contributed to the recovery of cyclophosphamide-induced alterations in interactions between the gut microbiota and immunity.

The gut microbiota modulates the anticancer immune effects of cyclophosphamide (33). Subsequent studies showed that antitumor efficacy of cyclophosphamide relies on two commensal species in the gut, *Enterococcus hirae* and *Barnesiella intestinihominis*, and these effects of the drug are limited by NOD2 receptors (34). Coincidentally, *Enterococcus faecalis* CGMCC0460.3, which is a strain belonging to the same genus as *Enterococcus hirae*, is a component of CBLEB; moreover, the NOD-like receptor signaling pathway was significantly downregulated in the spleen in the CTX+CBLEB group compared with that in the CTX group, suggesting that CBLEB may promote the antitumor effect of cyclophosphamide; these findings are worthy of additional studies.

CBLEB reversed overall disorder of gut metabolism induced by cyclophosphamide. In addition to direct contact, the gut microbiota can interact with the host through its metabolites (35). The results of the present study indicated that CBLEB reversed cyclophosphamide-induced alterations in the metabolome profile, including enrichment in 1-heptadecanol, succinic acid, hexadecane-1,2-diol, nonadecanoic acid, and pentadecanoic acid and depletion of benzenepropanoic acid and hexane. Human biosynthesis of these metabolites, with exception of succinic acid, has rarely been reported; therefore, alterations in the levels of all these compounds, even succinic acid, may be mainly related to alterations in the gut microbiota. Odd-numbered long-chain fatty acids, such as nonadecanoic acid and pentadecanoic acid, are primarily synthesized by microorganisms from propionic acid (36); pentadecanoic acid has various regulatory functions in cell signaling, glucose utilization, and maintenance of the integrity and stability of the gut epithelium. Fecal succinic acid is known as an intermediate for microbial synthesis of propionate and contributes to energy homeostasis (37). Benzenepropanoic acid is one of the adducts of phenylalanine decomposition by intestinal microbes (38), and the microbial metabolism pathways of hexadecane-1,2-diol and 1-heptadecanol are uncertain. These observations are in agreement with the results of correlation analysis of fecal metabolites and gut pathways obtained in the present study. For example, the results of the present study indicated that the gut protein digestion and absorption pathway was positively associated with fecal levels of hexane and negatively associated with fecal levels of nonadecanoic acid and succinic acid. Additionally, several studies used various analytical methods to demonstrate that cyclophosphamide can reduce the fecal levels of short-chain fatty acids beneficial to health (e.g., acetic acid, propionic acid, butyric acid, and valeric acid), and these effects are reversed after treatment with various agents, e.g., exopolysaccharides (26, 30). Detection of these compounds by different analytical methods may produce variable results (39); hence, these results in combination with the data of the present study more comprehensively describe the effects of cyclophosphamide and of the treatments by microbes or other substances on gut metabolism.

The results of the present study indicated that CBLEB partially restored cyclophosphamide-induced alterations in the serum metabolome. Blood is responsible for the transport of nutrients and metabolites throughout the body. CBLEB restored cyclophosphamide-induced enrichment in serum D-lyxose and depletion of serum succinic acid, D-galactose, L-5-oxoproline, L-alanine, and malic acid. D-lyxose is a rare pentose component of bacterial glycolipids and is not usually utilized by microbes (40). Succinic acid and malic acid are intermediate products produced in the biological tricarboxylic acid (TCA) cycle. Furthermore, in the serum, succinic acid plays cytokine-like roles, including promotion of the proliferation of hematopoietic cells mediated by phosphorylation of the ERK1/2 mitogen-activated protein kinase (MAPK) pathway and inositol phosphate accumulation in a pertussis toxin (PTX)-sensitive manner (41). Similarly, the results of the present study indicated that cyclophosphamide administration downregulated hematopoietic cell lineages, the MAPK signaling pathway, and the phosphatidylinositol signaling system in the spleen, and CBLEB administration reversed downregulation of the MAPK signaling pathway. Alanine is the second most abundant amino acid after glutamine in the circulation. Serum alanine is obtained from diet, transformation of glucose, or degradation of muscle protein during short-term starvation, acute exercise, or diseases (42). D-Galactose is a reducing sugar naturally present in the body and many foods and is important for human metabolism, including energy delivery and galactosylation (43). 5-Oxoproline is an important component of the γ-glutamyl cycle related to glutathione recycling, and glutathione levels are reduced due to conjugation with cyclophosphamide during detoxification (44). Therefore, alleviation of cyclophosphamide-induced dysbiosis of the serum metabolism by CBLEB partly reflects the mechanisms of the effects in cyclophosphamide-injected rats. A study used liquid chromatography and mass spectrometry to demonstrate that cyclophosphamide reduces the serum levels of lysophosphatidylcholine (LPC) (14:0), LPC (16:1), lysophosphatidylethanolamine (LPE) (18:2), LPC (22:6), and linoleic acid, which represents another aspect of the effect of cyclophosphamide on serum metabolism.

In addition to probiotics (27, 28, 45–47), some other orally administered agents, such as plant extracts, active components, and their derivatives, were shown to reduce immunotoxicity of cyclophosphamide in animals (48, 49). The effects of these substances are mediated by antioxidant and anti-inflammatory activities, protection of intestinal mucosal barrier, and maintenance of a balance of intestinal microbiota but are not limited to these mechanisms (48, 49). Nevertheless, the safety and effectiveness of most of these agents require further verification in humans. Intramuscular administration of rhG-CSF was reported to induce recovery from cyclophosphamide-induced leukopenia in mice (50). The study also identified cyclophosphamide-induced alterations in major signaling pathways in the spleen; however, the signaling pathways alleviated by CBLEB and rhG-CSF did not overlap. Specifically, rhG-CSF treatment alleviated cyclophosphamide-induced downregulation of cytokine-cytokine receptor interactions, the T cell receptor signaling pathway, and hematopoietic cell lineage and upregulation of the cell cycle, DNA replication, homologous recombination, mismatch repair, nucleotide excision repair, and pyrimidine metabolism. These findings correspond to potential differences in therapeutic mechanisms between rhG-CSF and CBLEB. The effects of rhG-CSF may be focused on the pathways related to DNA damage repair and metabolism of nucleotides and amino acids, and the effects of CBLEB may be focused on pathways related to signal transduction and on carbohydrate pathways; however, this conclusion is limited due to differences in animal models and methods.

In conclusion, CBLEB is a widely used probiotic drug that can significantly reverse immunodeficiency, alleviate systemic inflammation and metabolic dysbiosis, and restore the gut microbiota and metabolism in cyclophosphamide-injected rats. The mechanism of action of CBLEB is related to recovery of cyclophosphamide-altered carbohydrate metabolism and signal transduction. The present study provides an experimental foundation and comprehensive information on the application of CBLEB for the prevention and treatment of immunodeficiency, for promotion of the antitumor activity of cyclophosphamide, and for alleviation of toxicity and side effects of cyclophosphamide in the human body.

## Data Availability Statement

The datasets presented in this study can be found in NCBI Sequence Read Archive (SRA) with BioProject ID PRJNA721521.

## Ethics Statement

The animal study was reviewed and approved by Animal Care and Use Committee of the First Affiliated Hospital, School of Medicine, Zhejiang University.

## Author Contributions

LL, RY, and HJ conceived and designed the study. LL, DM, RY, and HJ performed the experiments and analyzed the data. LL and YD wrote the manuscript. All authors contributed to the article and approved the submitted version.

## Funding

This work is supported by the National Key Research and Development Program of China (no. 2018YFC2000500); the National Natural Science Foundation of China (nos. 81570512, 81790631, and 81790634); and the Natural Science Foundation of Zhejiang Province in China (no. LQ19H030007).

## Conflict of Interest

The authors declare that the research was conducted in the absence of any commercial or financial relationships that could be construed as a potential conflict of interest.

## Supplementary Material

Supplementary Figure 1 Representative changes in gene transcription in the colon and spleen verified by RT-qPCR. **(A)** Relative expression of *Tlr4* and *Tlr5* in the colon. **(B)** Relative expression of *Nf-κb* and *Tjp1*in the spleen. (*P < 0.05; **P < 0.01).
