# Supplementary figures and images for: Mechanism of the Immunomodulatory Effect of the Combination of Live Bifidobacterium, Lactobacillus, Enterococcus, and Bacillus on Immunocompromised Rats
Source: Front Immunol. 2021 Jun 15;12:694344. doi: 10.3389/fimmu.2021.694344 (PMC8239396; doi:10.3389/fimmu.2021.694344)

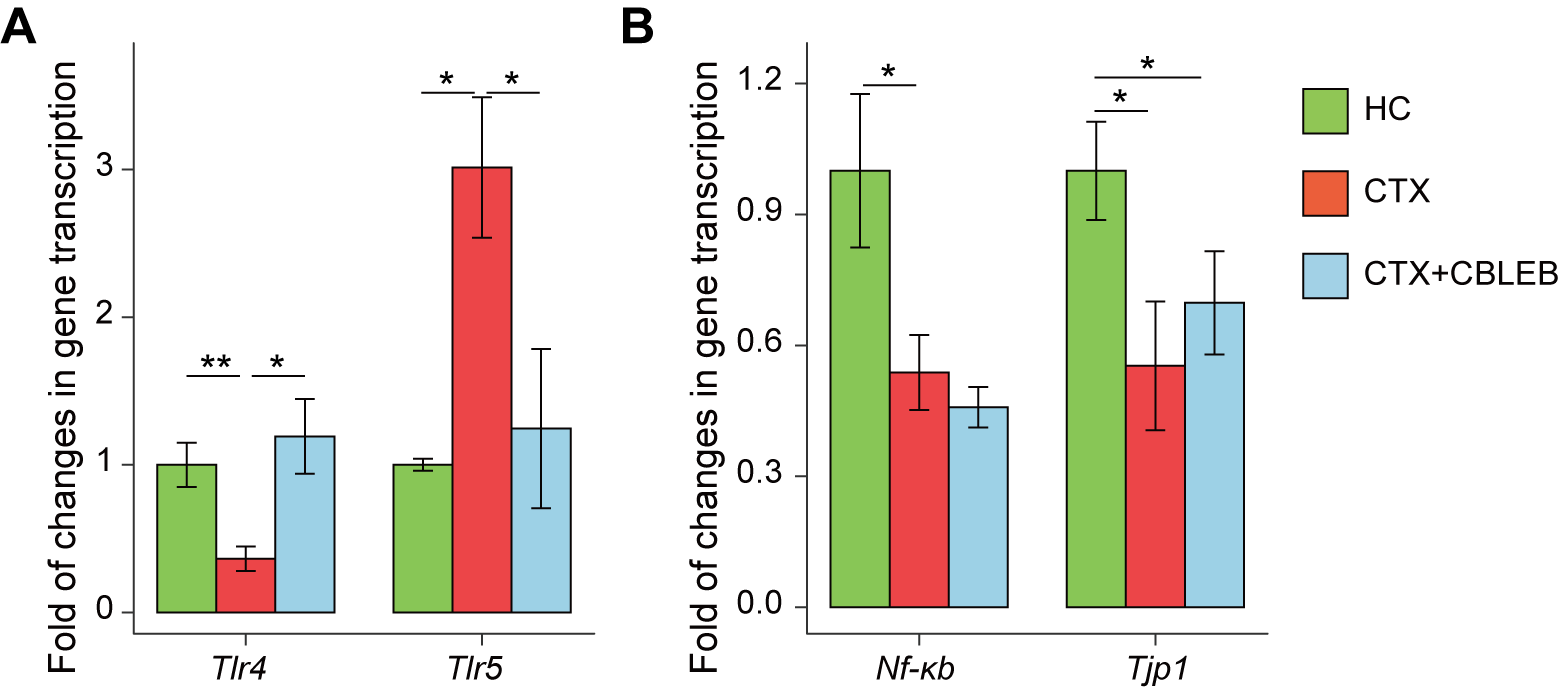

Supplement: Supplementary Figure 1 — Representative changes in gene transcription in the colon and spleen verified by RT-qPCR. (A) Relative expression of Tlr4 and Tlr5 in the colon. (B) Relative expression of Nf-κb and Tjp1in the spleen. (*P < 0.05; **P < 0.01). [file Image_1.tif]
